# Anti-Obesity Effect of Carotenoids: Direct Impact on Adipose Tissue and Adipose Tissue-Driven Indirect Effects

**DOI:** 10.3390/nu11071562

**Published:** 2019-07-11

**Authors:** Lourdes Mounien, Franck Tourniaire, Jean-Francois Landrier

**Affiliations:** 1 Aix Marseille Univ, INSERM, INRA, C2VN, 13385 Marseille, France; 2 CriBioM, criblage biologique Marseille, faculté de Médecine de la Timone, 13256 Marseille, France

**Keywords:** adipocytes, adipose tissue, brain, carotenoids, obesity

## Abstract

This review summarizes current knowledge on the biological relevance of carotenoids and some of their metabolites in obesity management. The relationship between carotenoids and obesity is considered in clinical studies and in preclinical studies. Adipose tissue is a key organ in obesity etiology and the main storage site for carotenoids. We thus first describe carotenoid metabolism in adipocyte and adipose tissue and the effects of carotenoids on biological processes in adipose tissue that may be linked to obesity management in in vitro and preclinical studies. It is also now well established that the brain is strongly involved in obesity processes. A section is accordingly devoted to the potential effect of carotenoids on obesity via their direct and/or adipose tissue-driven indirect biological effects on the brain.

## 1. Obesity, Comorbidities, Adipose Tissue and Brain Dysfunctions

The World Health Organization (WHO) defines obesity and being overweight as abnormal or excessive fat accumulation that presents a risk to health [1]. The risk is mainly related to comorbidities strongly linked to obesity such as metabolic inflammation, insulin resistance, liver steatosis, hypertension, dyslipidemia, certain types of cancer, depression, etc. The WHO states that in 2016, around 39% of the adult population were overweight, and about 13% of the world’s adult population were obese [1]. This prefigures a major public health issue in the short term not only in western countries but also in low- and middle-income countries, where an epidemic of obesity and being overweight is emerging.

The excess fat mass that characterizes obesity is produced by an expansion of adipose tissue mediated by hypertrophy and/or hyperplasia of adipocytes [2], which is linked to complex, tightly regulated adipogenesis. This process has been studied in depth, and both the temporal sequences and the transcriptional regulators involved have been identified. Among them, the nuclear receptor peroxisome proliferator-activated receptor gamma (PPARγ) and the CCAAT-enhancer-binding protein (CEBP) families are considered as transcriptional regulators of adipogenesis [3]. Through this mechanism, the adipose tissue can participate in energy homeostasis, allowing the storage of excess energy as triglycerides (lipogenesis) and the release of energy as fatty acids (lipolysis). This balance is tightly regulated, and dysregulation may result in body weight gain or loss.

Adipose tissue is also regarded as an endocrine tissue producing not only free fatty acids but also a wide variety of hormones, cytokines, chemokines and miRNA, together with adipokines and growth factors, acting on many physiological processes. Adipose tissue secretes approximately 50 biologically active proteins acting in an autocrine, paracrine and/or endocrine fashion. Leptin [4] and adiponectin [5] are among those most thoroughly studied. Both adipocytes and cells belonging to the stromal vascular fraction of adipose tissue, especially macrophages, are able to produce and secrete adipokines. Obesity triggers chronic low-grade inflammation associated with abnormal secretion of cytokines [6], chemokines [7], miRNA [8,9], acute phase proteins and other mediators of the immune response together with the activation of inflammatory signaling pathways [6,10]. Adipose tissue is a major contributor to the chronic inflammatory response. The regulation of substances secreted by adipose tissue is multifactorial and is linked to several pathophysiological disorders, including (i) increased levels of circulating free fatty acids, (ii) hypoxia of hypertrophied adipose tissue, (iii) systemic and local oxidative stress, (iv) endoplasmic reticulum stress and/or (v) the production of inflammatory cytokines. All these types of stress converge towards signaling pathways involving c-Jun amino-terminal kinase (JNK) and IκB kinase β (IKKβ) [6,10]. A large part of this inflammatory state is mediated by the increased number of infiltrated macrophages during expansion of adipose tissue [11]. This infiltration has been positively correlated with adiposity, adipocyte size and insulin resistance [12]. Macrophages interfere with adipocyte function through the production of pro-inflammatory cytokines such as tumoral necrosis factor α (TNF-α), interleukin (IL) 1β and IL-6. This can lead to insulin resistance, modified adipokine secretion and an excess of free fatty acid secretion through increased lipolysis and diminished lipogenesis [13], and therefore help install obesity-associated disorders such as insulin resistance.

Besides the effect of inflammatory state on adipocyte and adipose tissue function, it has also been shown that metabolic inflammation is associated with neuro-inflammation. Inflammation at the central level is widely suspected to be involved in obesity aetiologia via modulation of energy homeostasis (both at food intake and energy expenditure level) [14]. The control of energy homeostasis is finely tuned by nervous and endocrine mechanisms that cooperate to balance calorie intake and energy expenditure [15,16]. The central nervous system (CNS) continuously monitors modifications in hormones (insulin, leptin and ghrelin) or metabolic parameters (blood glucose or free fatty acids levels) and elicits adaptive responses, like food intake [15,16]. Among the brain regions involved in this regulation, the hypothalamus plays a pivotal role through specific neuronal networks [15,16,17,18]. In particular, leptin is crucial to maintaining both normal body weight and feeding behavior by action in the different regions of the hypothalamus such as arcuate, paraventricular or ventromedial nuclei, and the lateral hypothalamus. More specifically, this peripheral signal is detected by hypothalamic arcuate neurons expressing the anorexigenic peptide proopiomelanocortin (POMC) or the orexigenic peptides neuropeptide Y (NPY)/Agouti-related peptide (AgRP). These neurons project to melanocortin 3 and 4 receptor-expressing neurons located in the hypothalamus and other brain structures [16,19]. These neurons are collectively termed the melanocortin pathway, and regulate feeding behavior, energy expenditure and glucose homeostasis through the activation of the autonomic nervous system and higher brain structures [15,16,17]. A defect in the communication between brain and peripheral organs can affect fat gain and lead to metabolic syndrome.

Obesity leads to increased inflammatory factors and immune cells in peripheral tissues and in the brain regions that are essential for maintaining energy balance [14]. The production of inflammatory cytokines by adipose tissue and the activation of astrocytes and microglia (the resident immune cells of the brain) in the hypothalamus can interfere with leptin signaling and so contribute to hyperphagia and many other obesity-related diseases [15,16]. In this context, the endocrine function of the adipose tissue is essential to maintain normal weight and regulate energy homeostasis.

**Figure 1:**
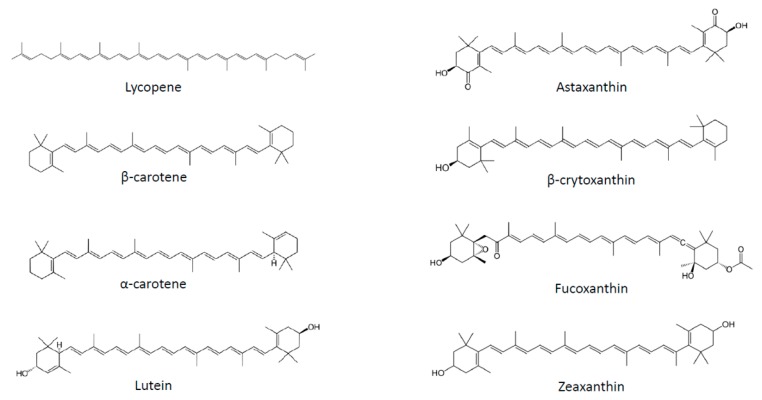
Chemical structures of the main carotenoids.

Several strategies have been proposed to fight obesity, including pharmacological approaches, limitation of fat and sugar consumption, promotion of physical activity and consumption of fruits and vegetables. Plant-based food is classically associated with weight management not only due to its macronutrient composition, but also to the presence of micronutrients, such as carotenoids. These substances correspond to a large family of C_40_ lipophilic pigments produced by plants, fungi and bacteria [20]. Carotenoids can be divided into two groups according to their chemical structure: carotenes, which are hydrocarbons, and xanthophylls, which also contain oxygen and are therefore less apolar than carotenes (Figure 1). More than 600 different substances have been identified, of which 50 can be found in the human diet, and of which only about 10 are present in significant amounts in human plasma [21]. Carotenoids containing an unsubstituted β-ionone ring are termed provitamin A, as they can be cleaved by animals to release retinal, which can subsequently be converted to retinol [20].

## 2. Carotenoids and Obesity in Human Studies

### 2.1. Observational Studies

Obesity has been associated in many epidemiological and observational studies with low circulating concentrations of carotenoids [22,23]. Strong inverse correlations between body mass index (BMI) and all measured carotenoids in plasma, except lycopene, were highlighted in the CARDIA study [24]. In addition, many obesity-associated disorders, such as low-grade inflammation or insulin resistance, are also strongly inversely associated with serum carotenoid concentrations [25,26,27].

### 2.2. Intervention Studies

Several trials have been conducted to study how carotenoids might be used in obesity management. Most of these studies used mixtures of carotenoids and vitamins in a natural matrix, such as fruit juices or plant extracts (reviewed by Bonet et al. [28]), making interpretation of the specific contribution of carotenoids difficult. To our knowledge, only two randomized double-blind placebo-controlled clinical trials have investigated the effect of pure carotenoid or xanthophyll supplementation. Canas et al. [29] reported a decrease in BMI *z*-score, waist-to-height ratio and subcutaneous adipose tissue in children given a mixture of carotenoids (β-carotene, α-carotene, lutein, zeaxanthin, lycopene, astaxanthin and γ-tocopherol) for 6 months. These beneficial effects were strongly associated with an increase in plasma β-carotene concentration in children [29]. Another study used a mixture of paprika xanthophylls and carotenoids, administered for 12 weeks to healthy overweight volunteers. This supplementation reduced visceral fat area, subcutaneous fat area and total fat area, along with BMI in the treated group compared to a placebo group [30].

## 3. Carotenoids and/or Metabolites are Involved in Body Weight Management and Limitation of Obesity Comorbidities in Preclinical Studies

Significant research has been devoted to studying the impact of β-carotene on energy metabolism and its outcome on obesity [31]. Its anti-obesity effect was subsequently demonstrated to be linked to a provitamin A effect [32,33], since β-carotene 15, 15′-monooxygenase (BCO) null mice did not display adipose tissue weight modification. This effect was found to be linked to decreased expression of PPARγ in adipose tissue and the involvement of retinoid X receptor (RAR) signaling in this regulation [34].

Astaxanthin prevented obesity in mice fed a high fat diet [35], via the limitation of adipose tissue expansion. Similar anti-obesity effects have been documented in mice fed a high fat and high fructose diet [36], where insulin sensitivity and inflammation were also improved by astaxanthin. Preventive effects of astaxanthin were found for hepatic steatosis [37] and inflammation and fibrosis in the liver in a non alcoholic steato hepatitis NASH and diet induced obesity (DIO) mice model [38].

Anti-adiposity properties have also been reported for β-cryptoxanthin [39], but their mechanism is still unknown. In addition, β-cryptoxanthin reversed liver steatosis and insulin resistance in DIO mice; this effect may be related to the anti-inflammatory effect of this carotenoid in the liver [40].

The potential of fucoxanthin for weight management has been extensively studied and reviewed [41]. This carotenoid limited weight gain and hyperglycemia, and inhibited the expression of several pro-inflammatory cytokines in adipose tissue of KK-a(y) mice [42]. Similar effects have been described in DIO mice, possibly through modulation of lipogenesis, adiponectin production and inflammation in adipose tissue [43], but also via browning of white adipose tissue [41].

Zeaxanthin inhibited obesity in high fat fed mice, presumably by inducing AMP-activated protein kinase (AMPK) activation, and inhibiting lipogenesis in adipose tissue [44].

The anti-obesity effect of lycopene was demonstrated in mice fed a high fat diet, where adiposity was reduced after supplementation. Several comorbidities were concomitantly reduced, such as glucose tolerance, insulin sensitivity and steatosis [45]. We and others have confirmed this beneficial effect of lycopene and/or tomato powder rich in lycopene in a DIO mice model on adiposity, glucose homeostasis, adipose tissue and liver inflammation and steatosis [46,47,48,49].

It is also clear that some of the effects of carotenoids (pro-vitamin A or other) are due to the vitamin A effect and are mediated by RAR. Such effects have been extensively reviewed elsewhere [28,31,50] and so will not be detailed here.

Most of these findings not only support the beneficial effect of several carotenoids on obesity management, but also strongly suggest that carotenoids may act on adipocyte/adipose tissue biology to modify several parameters linked to obesity and/or associated comorbidities. This hypothesis is strongly supported by the fact that carotenoids are stored and metabolized and are bioactive in adipocytes and in adipose tissue.

## 4. Carotenoids and Adipocyte/Adipose Tissue Metabolism

### 4.1. Carotenoids Are Stored in Adipocytes and Adipose Tissue

It has long been known that carotenoids are notably stored in adipose tissue [51,52,53,54,55]. Lycopene and β-carotene are the predominant carotenoids in human adipose tissue [53,56]. More precisely, Chung et al. identified lycopene as the most abundant carotenoid in adipose tissue (more than 1/2), followed by β-carotene (approx. 1/3 of total carotenoids), lutein + zeaxanthin, β-cryptoxanthin and α-carotene [54].

Total carotenoid concentration appears to be site-specific, with abdomen concentration higher than in the buttocks or thigh [54]. Adipose tissue concentrations of carotenoids are similar in men and women [54]. Interestingly, plasma levels of most carotenoids are inversely correlated to fat mass and to both general and central adiposity [54,57], suggesting that during obesity, carotenoids are sequestered in adipose tissue. Conversely, weight loss is associated with an increase in lutein and zeaxanthin serum concentration [58]. In the case of β-carotene, it is noteworthy that even if its adipose tissue concentration is lower in obese people, the total pool of β-carotene is similar in obese and non-obese when taking into account total fat mass [59].

Factors governing adipose tissue carotenoid uptake, distribution and turnover are poorly understood. However, we recently reported that carotenoid uptake by adipose tissue was independent of the carotenoid’s physical and chemical properties [60], suggesting the involvement of putative transporters or facilitators. Consistent with this, we demonstrated the involvement of a cluster of differentiation 36 (CD36) in lycopene and lutein uptake by adipose tissue and adipocytes [61]. We also showed that lycopene was mainly stored in lipid droplets in adipocytes, but was also present in plasma and nuclear membranes [62].

Adipose tissue carotenoid content is usually considered as a good long-term indicator of dietary intake of carotenoids [63]. β-Carotene concentration in adipose tissue increased 5 days after consumption of a large oral dose [64]. Lutein and zeaxanthin levels in adipose tissue significantly increased after spinach and corn supplementation in healthy subjects, with a maximum at 8 weeks of intervention [65]. Finally, tomato-oleoresin supplementation significantly increased lycopene concentration in adipose tissue [66]. Dietary carotenoid intakes were strongly correlated with abdomen adipose tissue concentration (a lower correlation was found for buttock or thigh adipose tissue) for α- and β-carotene, β-cryptoxanthin, cis-lycopene and total carotenoids [54]. However, these correlations vary widely and are strongly sex-related. Notably, El-Sohemy et al. reported correlation in women between intake and concentration in adipose tissues of α-, β-carotene, β-cryptoxanthin and lutein/zeaxanthin (CC 0.25, 0.29, 0.44 and 0.17, respectively), but not in men (CC 0.04, 0.07, 0.23 and 0.06, respectively) [67]. The origin of this discrepancy is presently unknown, but suggests that adipose tissue carotenoid concentration may be affected by factors other than intake, or that carotenoid intake is not appropriately estimated.

Adipose tissue carotenoid content is not only correlated with dietary intake, but also with other tissue concentrations. Thus, lutein adipose tissue content has also been reported to be positively correlated with macular pigment density in men, but not in women [68]. In addition, total carotenoid content in adipose tissue is strongly associated with serum levels [54], except for lycopene and lutein + zeaxanthin. β-Carotene content in adipose tissue is correlated with plasma level, with a correlation coefficient of 0.20 [63,69]. Similarly, breast adipose tissue carotenoid content correlates with plasma levels, except for β-cryptoxanthin [70].

### 4.2. Carotenoids Are Metabolized in Adipocytes and Adipose Tissue

BCO1, involved in centric cleavage of carotenoids and β-carotene 9′, 10′-dioxygenase (BCO2), involved in eccentric cleavage of carotenoids, are expressed in adipocytes [71], raising the possibility that carotenoid cleavage products, including retinal, derivatives and apocarotenoids, could be found in adipocytes [31,32,72]. In agreement, retinal [73] and free retinol have been identified in the adipocyte fraction of adipose tissue [74]. Several isomers of retinol, including all-trans, 9-cis and 13-cis isomers, were also quantified in white adipose tissue [74,75,76], together with several isomers of retinoic acid, except for 9-cis retinoic acid [75,77,78]. Adipocytes express BCO1 and BCO2, together with the enzymes necessary for vitamin A metabolism, suggesting that part of the effect of provitamin A carotenoids is mediated via vitamin A production. This topic will not be dealt with here; the reader is referred to the excellent review of Dr. Blaner [50].

Besides these retinoids, β-10′-apocarotenal has been identified in adipose tissue [32]. It is highly probable that other apocarotenoids are produced in adipose tissue, but their function in adipocyte biology needs further research.

### 4.3. Carotenoids Regulate Gene Expression in Adipocytes and Adipose Tissue

Several molecular mechanisms mediating the effects of carotenoids on gene expression have been described and may be related to the impact of carotenoids on adipocyte biology. In the case of provitamin A carotenoids, leading to retinoic acid synthesis, RARs and retinoid X receptors (RXRs), they constitute specific signaling targets. Two families of receptors mediate the effects of retinoids [79,80]. Three subtypes of each have been described (RARα RARβ, RARγ, RXRα, RXRβ and RXRγ). These receptors work as ligand-dependent transcriptional regulators by binding specific DNA sequences—retinoic acid response element (RARE) or retinoid X response element (RXRE)—found in the promoter region of retinoid target genes either as RAR-RXR or RXR-RXR dimers. RAR and RXR subtypes are found in every cell type. Furthermore, RXRs are dimerization partners for other nuclear receptors such as peroxisome proliferator activated receptors (PPARs), liver X receptor (LXR), farnesoid X receptor (FXR), pregnane X receptor (PXR), RARs, thyroid hormone receptor (TR) and vitamin D receptor (VDR), which are involved in the regulation of a huge number of genes. In addition, several other transcription factors and signaling pathways are modulated by retinoic acid [81], including PPARβ ([82]. Lycopene [83] and apo-10′-lycopenoic acid [84] are also able to activate RAR. Many carotenoids regulate gene expression via ubiquitous signaling pathways such as nuclear factor-kappa B (NF-κB) and mitogen activated proteins (MAP) kinases [85,86], or via transcription factors involved in detoxification such as aryl hydrocarbon receptor (AhR), nuclear factor erythroid-2-related factor 2 (NRF2) or PXR [87,88].

## 5. Carotenoids and/or Metabolites Impact Adipocyte Biology In Vitro Studies

**Figure 2:**
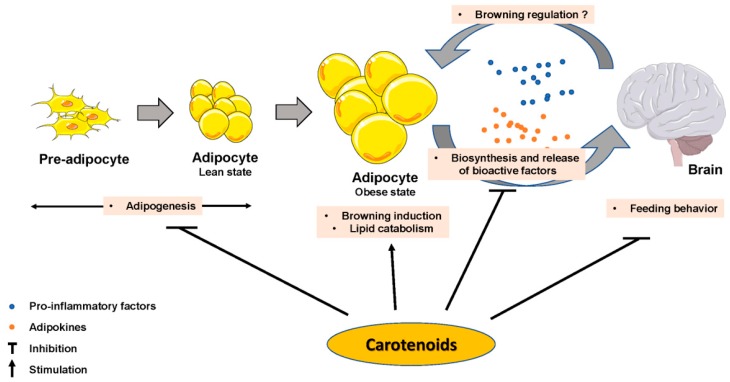
Carotenoids effect on adipose tissue biology parameters, on brain and on adipose tissue–brain crosstalk.

The impact of some carotenoids has been documented in adipogenesis (Figure 2), which could help obesity management via a limitation of lipid accumulation in adipocytes. Most of the reported effects inhibited adipocyte differentiation [89] by interfering with nuclear receptors such as RAR, RXR or PPAR. β-Carotene inhibited adipogenesis through the production of β-apo-14′-carotenal and repression of PPARα, PPARγ and RXR activation [90], but also through the production of all-trans retinoic acid [34]. Similarly, β-cryptoxanthin suppressed adipogenesis via activation of RAR [91], and astaxanthin inhibited rosiglitazone-induced adipocyte differentiation by antagonizing transcriptional activity of PPARγ [92]. Zeaxanthin [44] and fucoxanthin [93,94] exhibited anti-adipogenic effects via a down-regulation of adipogenic transcription factors C/EBPα and PPARγ, which blunted lipid accumulation. Conversely, lycopene (unpublished personal data) and apo-10′-lycopenoic acid [84] showed no effect on adipogenesis. Besides these effects, there is evidence that some effects of provitamin A carotenoids are mediated through retinol and its metabolite production, which are known to regulate adipogenesis [50].

Substances with anti-inflammatory effects are assumed to limit the risk of obesity-associated disorders, including insulin resistance. Such anti-inflammatory effects of β-carotene in 3T3-L1 adipocytes were suggested to arise through limitation of the TNFα-mediated down-regulation of genes linked to adipocyte biology [95]. β-Carotene also counteracted oxidative stress-mediated dysregulation of adiponectin secretion, chemokine expression and NF-κB activation in 3T3-L1 adipocytes [96]. Fucoxanthin blunted TNFα-mediated induction of pro-inflammatory cytokines in adipocytes [42] and in adipocyte/macrophage coculture systems [97]. The most thoroughly studied anti-inflammatory carotenoid is lycopene (all-trans), and we demonstrated its ability to inhibit proinflammatory cytokine and chemokine expression in vitro (in murine and human adipocytes) [98]. These data were also reproduced ex vivo on adipose tissue explants from mice fed a high fat diet (characterized by low-grade inflammation). The molecular mechanism was investigated and the involvement of NF-κB was confirmed. Similar results (inhibition of cytokine and chemokine expression in various in vitro and ex vivo models) were obtained with apo-10′-lycopenoic acid, a metabolite of lycopene [84]. Lycopene also attenuated LPS-mediated induction of TNFα in macrophages via NF-κB and JNK [99], as well as macrophage migration in vitro. Consequently, lycopene decreased macrophage-induced cytokine, acute phase protein and chemokine mRNA in adipocytes. Interestingly, all-trans and 5-cis lycopene, the two main isoforms of lycopene found in vivo, displayed similar effects in terms of inflammation control and glucose uptake in adipocytes [100]. A few studies have shown that retinoids, like carotenoids, have positive effects by decreasing the expression of adipocyte-derived inflammatory mediators such as adipsin [101] and resistin [102]. Our group has also shown that all-trans retinoic acid (ATRA) blunts TNF-α mediated cytokine expression in 3T3-L1 cells [84]. More recently, we demonstrated that ATRA limits the expression of a large range of chemokines in vivo and in vitro. This anti-inflammatory effect of ATRA was associated with a reduction in the phosphorylation levels of IκB and p65, probably mediated by peroxisome proliferator-activated receptor gamma coactivator 1 α (PGC1α) expression [103].

The browning of white adipose tissue has been proposed as a putative mechanism controlling energy homeostasis and insulin sensitivity [104]. Recently, an AMPK-mediated effect on adipocyte browning and mitochondrial biogenesis was demonstrated for zeaxanthin [105] and for lycopene [49]. We reported similar mitochondrial biogenesis, induction of oxidative phosphorylation (OXPHOS) and adipocyte browning in adipocytes incubated with ATRA [106], whereas fucoxanthin and its metabolite fucoxanthinol were inefficient in inducing adipocyte browning [107].

Taken together, these findings suggest that carotenoids impact several adipocyte metabolic pathways, which may in turn explain, at least in part, their anti-obesity effects (Figure 2).

## 6. Impact of Carotenoids on the Control of Energy Homeostasis by the Brain

As stated above, carotenoids can affect the biology of the adipose tissue and modulate the production of leptin and the inflammatory cytokines [55]. They may consequently have an indirect effect on brain function (Figure 2). However, several food components, including carotenoids, could reach the hypothalamus directly [108], where they could regulate leptin signaling pathways. To support this hypothesis, several carotenoids have been detected in several parts of the adult brain [109,110]. In the study of Johnson et al., lycopene (37 +/− 9 pmol/g), lutein (145 +/− 22 pmol/g), β-carotene (77.6 +/− 10.5 pmol/g) and zeaxanthin (45 +/− 7.5 pmol/g) have been quantified in different structures (cerebellum, frontal, occipital and temporal cortices). More specifically, they could either cross the blood brain barrier or pass through the fenestrated capillaries of circumventricular organs and target the arcuate nucleus neurons. In the context of the central control of feeding behavior, it is important to note that other structures such as the hippocampus play an important role and that they could be targeted by carotenoids as indicated below.

It is presently not clear whether carotenoids act indirectly via adipose tissue or directly on the brain, but several studies suggest involvement of the brain in body weight management under the effect of carotenoids. Continuous intake of lycopene-rich food and intraperitoneal administration of lycopene increased neuronal activity in the paraventricular and ventromedial nuclei, as shown by the immunoreactivity of c-fos, a marker of neuronal activity [111]. This study suggests that lycopene may have some influence on feeding behavior. In support of this hypothesis, the group of Dr. Bishnoi showed that lycopene prevented weight gain and adiposity in mice in a DIO model [45]. Interestingly, this effect was associated with a modulation of hypothalamic anorexigenic and orexigenic gene expression. To date, the direct effect of lycopene on neuronal activity is unclear, and more research is needed to thoroughly understand this mechanism. As stated above, lycopene can impact brain function by limiting peripheral inflammation. In support of this hypothesis, Kuhad et al. showed that chronic treatment with lycopene significantly and dose-dependently attenuated cognitive deficit associated with inflammation in diabetic rats [112].

Interestingly, recent work by Zhao et al. suggests that fucoxanthin may modulate neuroinflammation [113]. In this work, fucoxanthin increased NRF-2 activation in lipopolysaccharide (LPS)-activated microglia. This interesting effect needs to be studied in an in vivo model and especially in brain structures involved in feeding behavior (i.e., hypothalamus or hippocampus). In the same line, a recent paper reported that fucoxanthin treatment reversed LPS-induced defect in body weight and food intake in mice [114]. The authors also showed that fucoxanthin inhibited LPS-induced overexpression of pro-inflammatory cytokines (IL-1β, IL-6 and TNF-α) in the hippocampus and hypothalamus, via the modulation of the AMPK-NF-κB signaling pathway. Interestingly, current studies have shown that the activation of the AMPK pathway is essential to maintaining energy homeostasis, as it is involved in the anorexigenic effect of leptin [115].

## 7. Conclusions

In vitro and preclinical studies clearly indicate beneficial effects of carotenoid consumption on obesity and associated pathophysiological disorders including metabolic inflammation, insulin resistance and hepatic steatosis. Molecular mechanisms are now better known, although it is not always clear whether carotenoids are active in their native form or after cleavage and metabolization, and adipose tissue appears as a major target of these substances. Nevertheless, recent though limited data suggest that carotenoids or metabolites might also act at the central level, probably by preventing or decreasing obesity-associated neuro-inflammation and comorbidities. Randomized clinical trials using pure carotenoids are urgently needed to support preclinical and observational evidence.
